# Lignocellulosic Byproducts as Bio-Adsorbents for Lead Removal

**DOI:** 10.3390/ma18102320

**Published:** 2025-05-16

**Authors:** Morgana Macena, Helena Pereira, Lucas Grosche, Bruno Esteves, Isabel Santos-Vieira, Luísa Cruz-Lopes

**Affiliations:** 1 CERNAS Research Centre, Polytechnic Institute of Viseu, 3504-510 Viseu, Portugal; bruno@estgv.ipv.pt; 2 CEF—Forest Research Centre, School of Agriculture, University of Lisbon, 1349-017 Lisboa, Portugal; hpereira@isa.ulisboa.pt; 3 4iTec Lusitânia S.A., Lugar do Pombal, Zona Industrial do Salgueiro, 3530-259 Mangualde, Portugal; lucas.grosche@4iteclusitania.pt; 4 CICECO—Aveiro Institute of Materials, Department of Chemistry, University of Aveiro, 3810-193 Aveiro, Portugal; ivieira@ua.pt

**Keywords:** lead removal, lignocellulosic bio-adsorbents, adsorbent characterization, adsorption mechanisms

## Abstract

Water pollution by toxic metals, especially by lead ions, is a serious environmental concern due to these metals’ persistence, bioaccumulation, and toxicity. Aiming to reduce metal concentrations to non-toxic levels, this study analyzed the removal of lead from water through adsorption with bio-adsorbents. The adsorbent potential of the following four lignocellulosic byproducts were tested: walnut and chestnut shells, pine wood, and burnt pine wood. Removal rates of 97–99% were achieved at optimized conditions, i.e., at a pH of approximately 7, adsorbent dose of 4 g L^−1^, and 12 h of reaction. The BET specific surface area was between 1.74 and 4.85 m^2^ g^−1^. The pore size of the wood bio-adsorbent was 26.54 nm, and those of the remaining materials were between 5.40 and 7.33 nm. With R^2^ = 0.998–1.000, the kinetics fit the pseudo-second-order model better, suggesting that chemisorption is the dominant mechanism. Both Langmuir and Freundlich isothermal models fit the data well, with R^2^ = 0.946–0.999. It can be concluded that all the bio-adsorbents tested have the potential to efficiently remove lead ions from water.

## 1. Introduction

Water pollution by toxic metals has increased due to industrialization and the expansion of new technologies, thereby becoming a serious environmental concern given these metals’ persistence, non-biodegradability, ease of bioaccumulation, and lethal toxicity [1,2]. According to the European Environment Agency [3], the heavy metals that are usually found in water are cadmium (Cd), lead (Pb), mercury (Hg), and nickel (Ni), which have negative impacts on the environment and human health.

Lead is widely used for industrial purposes such as battery manufacturing, painting, the coating of electrical cables, metal plating, and mineralization [4]. The World Health Organization has classified lead as one of the most dangerous chemical elements for humans and animals [5,6]. This metal has the particularity of accumulating in humans’ tissues and affects the nervous system, disrupts the normal metabolic functioning of the body, and is potentially carcinogenic [7,8]. The exposure to lead can also disrupt brain development in children and affect the male reproductive system. Lead is also toxic to plants and animals [8,9].

Human contact with lead can happen through drinking water, which is predominantly contaminated by lead plumbing, or due to various industrial processes [10,11]. Groundwater can be contaminated with lead from the excessive use of agrochemicals and industrial effluents discharge, as many reports have shown. For instance, Pb levels of 0.194–0.287 ppm and 0.005–0.41 ppm were found in groundwater samples from India and Pakistan, respectively [12,13], and Pb concentrations of 2.064 ppm and 0.77 ppm were measured in rivers in Peru and Ecuador, respectively [14]. These values are much higher than the limit of 15 ppb of Pb in drinking water established by the EPA [7], or the limit of 0.01 and 0.1 ppm of Pb in wastewater and soils used for agriculture, respectively, established by WHO [15].

Because of restrictive legislation, there is a demand for new technologies that can successfully remove pollutants from water, and also for integrating the restoration of contaminated areas or water sources through products and processes that reduce the generation of harmful substances [16]. The reuse of treated water can offer a significant increase in the amount of water available, thereby dealing with the insufficiency or poor quality of water resources [3,17].

The conventional treatments for removing metal ions from water are chemical reduction, ion exchange, ultrafiltration, flocculation, biological degradation, and reverse osmosis. In most cases, these are expensive processes that generate byproducts that need to be treated in another step of processing [18,19]. Adsorption arises as an efficient and easy-to-operate method [19].

Many studies have focused on low-cost biological adsorbents, such as lignocellulosic wastes, to efficiently capture metals from water because they are sustainable materials and available in large quantities [20,21,22,23,24]. Several adsorbents, such as rice straw [25], coconut shells [26], peanut shells [27], coffee husks [28], oranges, lemons, bananas, and watermelon peels [29], have been tested for Pb removal from water. Lignocellulosic wastes from agricultural and forest exploitation consist mainly of cellulose, hemicelluloses, and lignin, which have several functional groups that can bind the lead from aqueous solutions [30].

The purpose of this study was to verify the efficiency of removing lead from water by adsorption under optimal pH conditions using different lignocellulosic wastes: walnut shell, chestnut shell, wood, and burnt wood (*Pinus pinaster*). The evaluation of the adsorption mechanisms was performed through kinetic and isothermal models. The morphological analysis of the adsorbent material was also performed using scanning electron microscopy (SEM), the Brunauer–Emmett–Teller (BET) method, and powder X-ray diffraction analysis (PXRD).

## 2. Materials and Methods

### 2.1. Materials

The lignocellulosic waste materials tested as bio-adsorbents were obtained in the region of Viseu, central Portugal, and consisted of the following:

- Dry nut shells of walnut and chestnut obtained as waste material at one dry fruit processing mill, and
- Wood and burnt wood obtained from local pine (*Pinus pinaster*) forests. Burnt pine wood is abundant due to wildfires that have occurred in the region in the last years.

Before milling and sieving, the materials were dried at 60 °C in an oven to eliminate excess moisture. The granulometry used for the adsorption tests was <80 mesh (<0.177 mm), because the smaller the particle size, the greater its specific surface area available to retain metal ions. The adsorbent materials were dried at 105 °C for 24 h to completely remove moisture content.

A stock solution of lead (Pb(NO_3_)_2_ in distilled water) was prepared to be used in the adsorption batch experiments at the desired concentration (5, 10, 15, 25, 50, 75, 100, 150, and 200 mg L^−1^).

### 2.2. Adsorbent Material Characterization

#### 2.2.1. Powder X-Ray Diffraction (PXRD) Analysis

The crystalline structure of the samples was analyzed by X-ray diffraction (PXRD) using a Philips X’Pert MPD diffractometer (Malvern Panalytical B.V., Lelyweg 1, 7602 EA Almelo, The Netherlands) equipped with a Cu Kα radiation source (λ = 0.154 nm) and a Ni filter, operated at 40 kW and 20 mA. Scans were performed in the 2θ range of 2–40°, with a scanning step size of 0.02° and a scan rate of 2° min^−1^.

#### 2.2.2. Scanning Electron Microscopy and Energy Dispersive Spectroscopy (SEM–EDS)

The morphology of the powder samples was evaluated by scanning electron microscopy (SEM) using a Hitachi S-4100 microscope (Hitachi High-Tech Corporation, 1-24-14 Nishi-Shimbashi, Minato-ku, Tokyo 105-8717, Japan), operated at 20 kV. Prior to analysis, samples were gold-coated to ensure conductivity. Elemental composition was further assessed by energy dispersive X-ray spectroscopy (EDS).

#### 2.2.3. BET Surface Area Analysis

The specific surface areas and porosities of the samples were determined with N_2_ adsorption–desorption isotherms using the Brunauer–Emmett–Teller (BET) method.

#### 2.2.4. Thermogravimetric Analysis

In a comprehensive examination of the thermal degradation behavior related to the presence of lead in a sample, thermogravimetric analysis (TGA) was employed to determine weight loss following a rise in temperature. The mass loss was monitored throughout the entire heating process, which occurred at a rate of 5 °C min^−1^, up to 800 °C. The analysis was carried out using a Mettler V3 thermobalance coupled to a Mettler TC10A processor (Mettler-Toledo AG, Im Langacher 44, 8606 Greifensee, Switzerland).

### 2.3. pH Optimization

**Figure 1:**
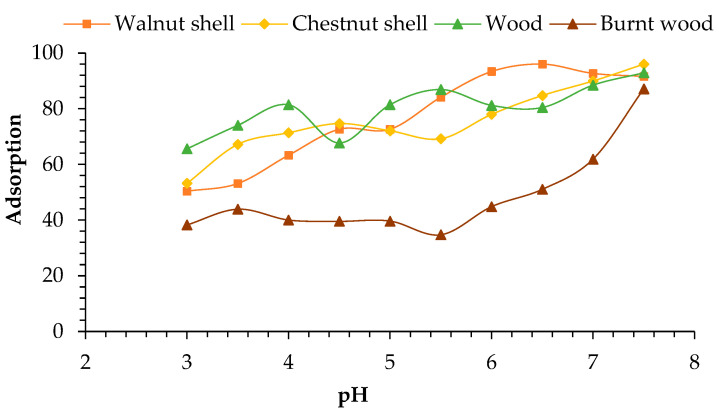
Removal of lead ions (%) at different pH conditions. Adapted from [31].

In a previous study, the ideal pH to remove lead, nickel, and chromium from water using walnut and chestnut shells, pine wood, and burnt pine wood, was tested at a pH range of 3 to 7.5 [31]. All bio-adsorbents were efficient in removing lead ions, as demonstrated in Figure 1, with maximal removal around 96% for chestnut shells, 93% for wood, and 87% for burnt wood at pH 7.5, and 96% for walnut shells at pH 6.5 [31]. Thus, in this study, the adsorption tests were carried out at pH 7.5 for chestnut shells, wood, and burnt wood and at pH 6.5 for walnut shells.

### 2.4. Adsorption Isotherms

The effect of the initial concentration of Pb in the adsorption was evaluated. Adsorption tests were performed at the optimized pH values, with concentrations of lead varying from 5 to 200 mg L^−1^, an adsorbent dose of 4 g L^−1^, and 12 h of agitation. The removal rate was calculated based on the remaining concentrations measured by atomic absorption spectroscopy (Perkin Elmer AAnalyst 300, PerkinElmer U.S. LLC 710 Bridgeport Avenue, Shelton, CT 06484-4794, USA). All adsorption tests were performed at room temperature, in triplicate.

The isotherm expresses the relationship between the concentration of adsorbate and its accumulation on the surface of the adsorbent [20]. The most-used models to characterize the isotherm of the reaction are the Langmuir and Freundlich models. The Langmuir isotherm assumes surface homogeneity and monolayer surface coverage [29], while the Freundlich model assume heterogeneous surfaces and multilayer sorption [16]. Both linearized models (Equations (1) and (2)) were evaluated in this study.

The Langmuir equation isotherm (Equation (1)) is represented by the following equation:

(1) $$\frac{1}{q_{e}}=\frac{1}{q_{max}}+\left(\frac{1}{K_{L}q_{max}}\right)\times \frac{1}{C_{e}}$$

where *qe* expressed in mg g^−1^ represents the quantity adsorbed (Pb) in each g of adsorbent material, *qmax* corresponds to the saturation capacity of the adsorbent material, *Ce* in mg/L is the equilibrium concentration of the adsorbate, and *KL* is the Langmuir constant.

The Freundlich equation isotherm (Equation (2)) is represented as follows:

(2) $$\log q_{e}=\log K_{F}+\frac{1}{n}\log C_{e}$$

where *qe* is the amount of metal adsorbed per unit mass of adsorbent (mg g^−1^). By plotting log *qe* vs. log *Ce*, which is the concentration of metal at equilibrium (mg L^−1^), we can determine the *n* parameter by the slope of the linear regression, which indicates the magnitude of the surface heterogeneity.

### 2.5. Adsorption Kinetics

The kinetics describes the rate of sorption on the surface of the material, providing information on the reaction mechanisms [32]. The most popular kinetic models are the pseudo-first-order (PFO) and pseudo-second-order (PSO) models [20]. In this study, the Elovich and intraparticle diffusion models were also considered. The kinetics were evaluated by making adsorption tests with 25 mg L^−1^ lead solutions at optimized pH and with time agitation from 10 to 1440 min.

The distribution coefficient (*Kd*) was calculated as follows:

(3) $$K_{d}=\frac{C_{0}-C_{e}}{C_{e}}\times \frac{V}{m}$$

where *C*0 (mg L^−1^) is the initial concentration of metal, *Ce* (mg L^−1^) is the concentration of metal ions when adsorption equilibrium is reached, *m* (g) is the weight of the adsorbent, and *V* (L) is the volume of the adsorbate.

The mathematical equations used to determinate the kinetics for the PFO (Equation (4)), PSO (Equation (5)), Elovich (Equation (6)), and intraparticle diffusion models (Equation (7)) are expressed below:

(4) $$\ln(q_{e}-q_{t})=\ln q_{e}-k_{1}t$$

(5) $$\frac{t}{q_{t}}=\frac{1}{k_{2}q_{e}^{2}}+\frac{t}{q_{e}}$$

(6) $$q=\left(\frac{1}{b}\right)\ln\left(ab\right)+\left(\frac{1}{b}\right)lnt$$

(7) qt=kintt12+C

where *qt* (mg g^−1^) is the adsorbed quantity at time *t*; *qe* (mg g^−1^) is the adsorbed metal quantity at equilibrium; *k*1 (min), *k*2 (g mg^−1^.min), and *kint* (mg g^−1^.min^1/2^) are the corresponding adsorption rate constants; *a* (mg.g min^−1^) is the initial sorption rate constant; and the parameter *b* (g mg^−1^) is related to the extent of surface coverage and activation energy for chemisorption.

## 3. Results

### 3.1. Characterization of Materials

Walnut shells exhibited the highest BET surface area (4.855 m^2^ g^−1^), followed closely by chestnut shells (4.210 m^2^ g^−1^). Both materials also presented relatively large pore volumes, 1.195 × 10^−3^ and 4.202 × 10^−3^, respectively, compared to wood (4.920 × 10^−4^), indicating their potential for enhanced adsorption capabilities. Conversely, wood displayed the lowest surface area (1.738 m^2^ g^−1^) and the largest average pore size (26.54 nm), suggesting a more macroporous structure with a reduced number of adsorption-active sites.

**Figure 2:**
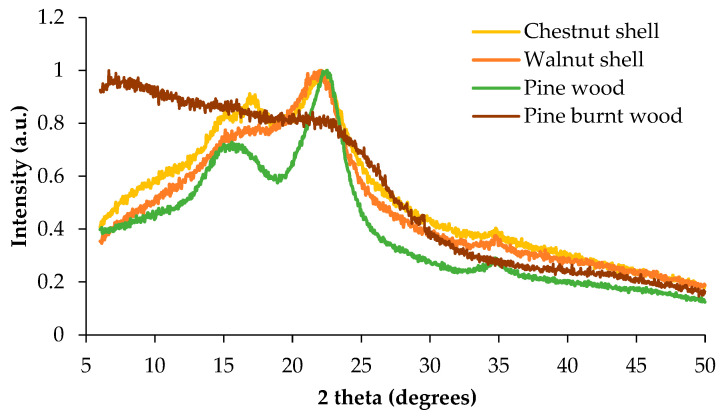
Powder X-ray diffraction of chestnut and walnut shells, wood, and burnt wood.

PXRD analysis was also performed, and the patterns are presented in Figure 2. None of the materials exhibited sharp diffraction peaks, confirming the predominantly amorphous nature of the samples. The presence of two broad diffraction bands around 16° and 22° suggests the contribution of cellulose in a semi-crystalline state [33].

**Figure 3:**
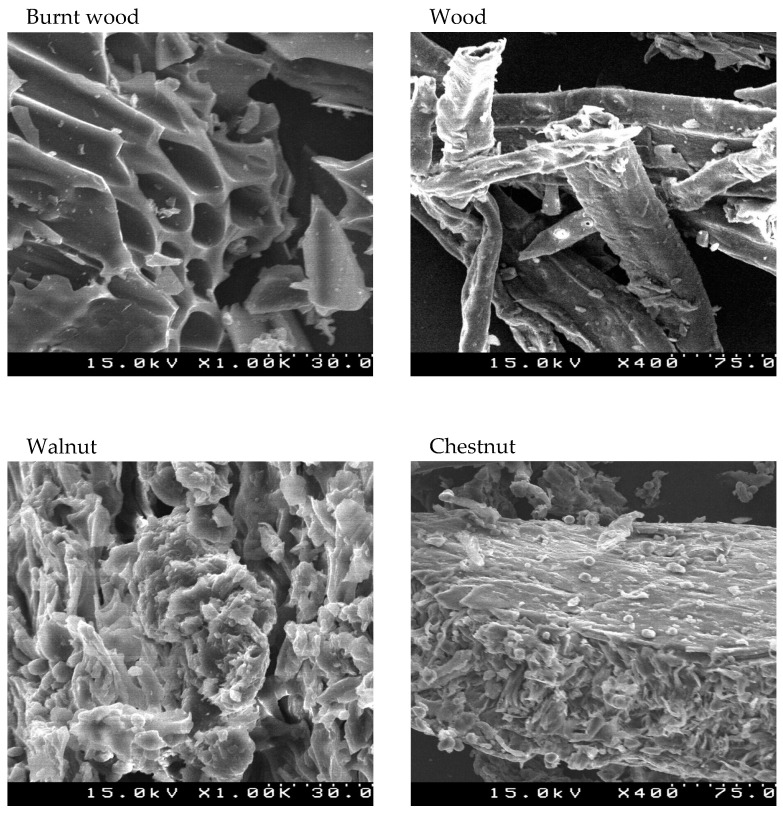
SEM images of burnt wood, pine wood, and walnut and chestnut shells. The burnt wood and walnut images have a magnification of 1000×, and the wood and chestnut images have a magnification of 400×.

The morphology of the different samples was analyzed using SEM images (Figure 3), which do not reveal distinctive morphological features but allow for a quantitative comparison of the average observed sizes. The chestnut sample exhibited an average size of 0.48 cm on the macroscopic scale and 6.54 µm on the microscopic scale. The walnut sample exhibited a larger macroscopic average size (0.62 cm) but a smaller microscopic size (3.38 µm), suggesting a possibly more compact or homogeneous structure. The wood sample had average sizes of 0.58 cm and 7.90 µm, falling between the values recorded for chestnut and walnut. The burnt wood sample stood out with the largest average size, both macroscopically (1.92 cm) and microscopically (10.47 µm), which may indicate significant structural changes due to the combustion process leading to a more porous or fragmented matrix. These results suggest that the observed differences among the samples may be related to their composition and internal structure, and influenced by factors such as density, porosity, and thermal transformations.

EDX experiments were also conducted to assess the absorption of Pb in the samples; however, due to the very low concentrations, it was not possible to detect this using EDX. To complement these experiments, attempts were made to perform thermogravimetric and SEM analyses, as seen in Figure 3, but once again, the quantities were too small to accurately quantify the amounts of Pb.

### 3.2. Initial Concentration of Pb Ions

**Figure 4:**
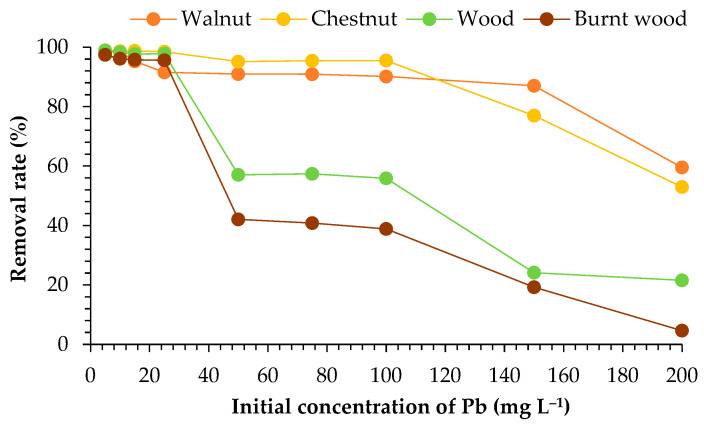
Removal rate of Pb (%) under different initial concentrations of ions in the solution (mg L^−1^).

The results found for Pb removal at different initial concentrations are presented in Figure 4. Adsorption rates of almost 100% were achieved for all the materials tested at the lowest initial concentration (5 mg L^−1^). The adsorption rates were 60–99% for walnut, 53–99% for chestnut, 22–99% for wood, and 5–97% for burnt wood. There was a tendency for the removal rate to reduce with an increase in the initial concentration of Pb ions. For walnut and chestnut, a sharp decrease was noted at 150 mg L^−1^. For wood and burnt wood, the decrease in the removal was noted at 50 mg L^−1^, with rates of 57 and 42%, respectively, reducing even more after that.

### 3.3. Adsorption Isotherms

**Figure 5:**
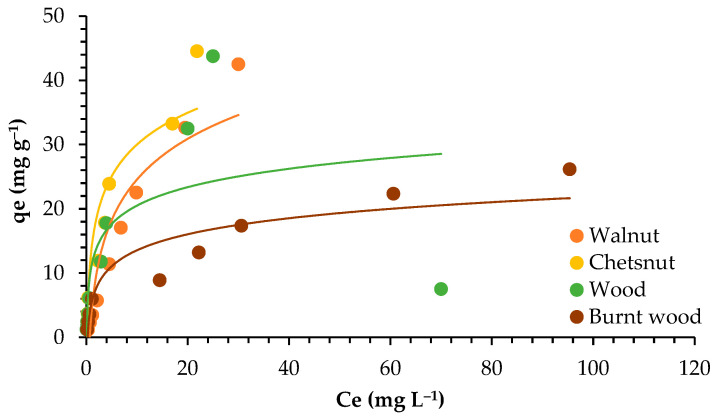
Pb ions adsorbed (mg g^−1^) in relation to Pb concentration in the solution (mg L^−1^) at equilibrium.

The isotherm describes how the interaction between the adsorbent surface and the adsorbate occurs. It is highly dependent on the kind of adsorbent used. The efficiency of adsorption is dependent on the concentration of ions in the solution and the availability of active sites to bind the ions. Figure 5 presents the plotting of the adsorbed Pb ions (*qe*) and the concentration of Pb (*Ce*) in solution at equilibrium.

**Figure 6:**
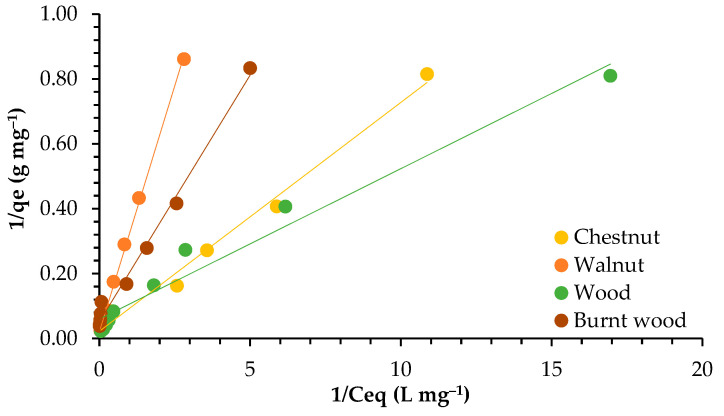
Adsorption isotherms of lead for walnut shell, chestnut shell, wood, and burnt wood in Langmuir linearized form.

**Figure 7:**
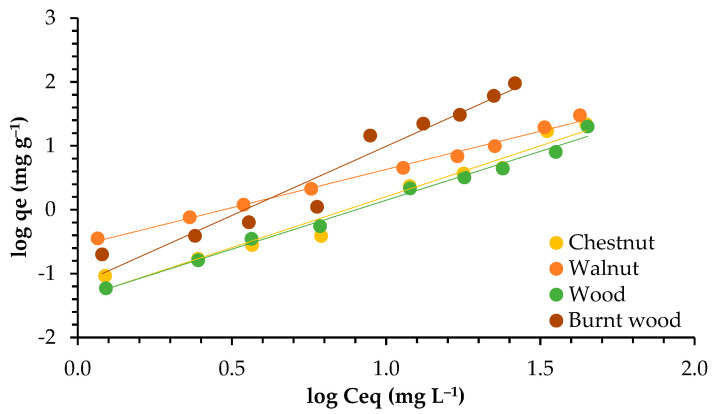
Adsorption isotherms of lead for walnut shell, chestnut shell, wood, and burnt wood in Freundlich linearized form.

In Figure 6 and Figure 7, the plotting of Langmuir and Freundlich linearized isotherm models is presented.

The correlation coefficients (R^2^), K_L_ and K_F_ constants, and estimated *qmax* and *n* from the Langmuir and Freundlich models for all materials tested are present in Table 1.

Both models worked well to describe the adsorption isotherm, according to the R^2^ values, which were between 0.946 and 0.999. However, the Langmuir model best explained the adsorption of lead for walnut shell, chestnut shell, and burnt wood, with R^2^ values from 0.991 to 0.999. For wood, on the other hand, the Freundlich model fit better, with R^2^ = 0.991.

The Freundlich constant *n* presented values greater than 1, indicating a favorable adsorption process for all adsorbents, given that *n* values ranging from 1 to 10 indicate stronger interaction between biosorbent and metal. Higher K_F_ values of 7.483 and 8.002 were found for chestnut shell and wood, respectively, compared to walnut shell (2.990) and burnt wood (3.621).

The *qmax* values were in the range 16.639–47.393 mg g^−1^, indicating that good adsorption was achieved. The highest value was obtained for walnut, showing that walnut can accommodate a higher amount of lead in its surface. This could also be related to the fact that it has the highest surface area (4.855 m^2^ g^−1^).

### 3.4. Adsorption Kinetics

**Figure 8:**
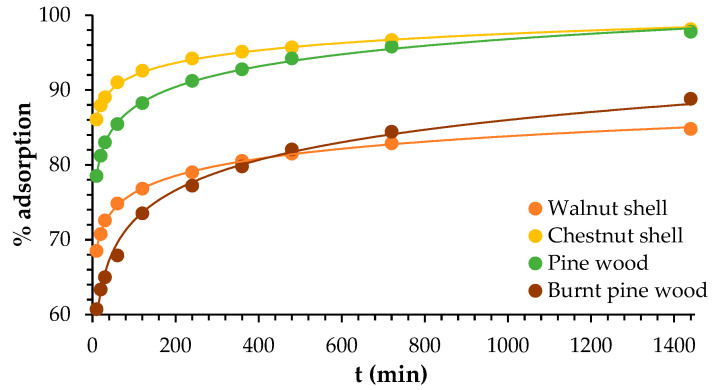
Adsorption rate of Pb (%) as a function of time (min).

**Figure 9:**
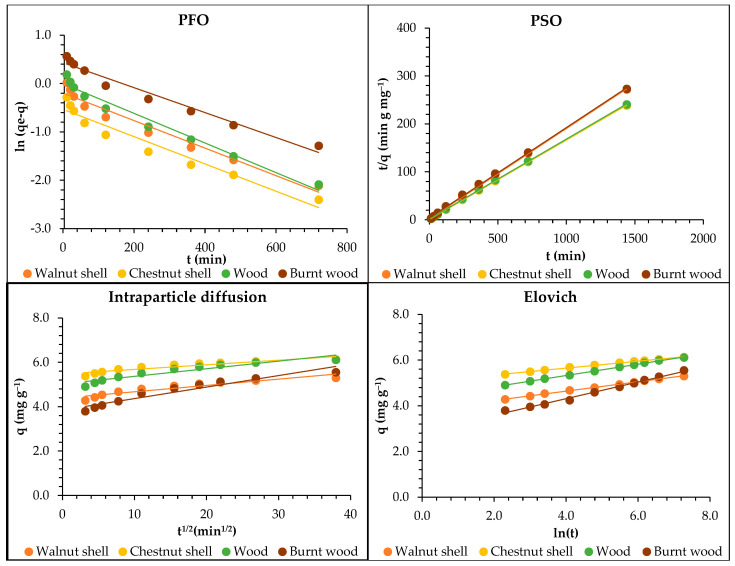
Graphical representations of the kinetic models.

The representation of the adsorption rate (%) of Pb ions as a function of time (min) is presented in Figure 8. In Table 2 and Table 3, the kinetic parameters for Pb adsorption onto walnut shell, chestnut shell, wood, and burnt wood are presented for the PFO, PSO, Elovich, and intraparticle diffusion models, respectively. Graphical representations of the kinetic models tested are presented in Figure 9.

According to the R^2^ found, the model that best explained the kinetics process was the PSO model. However, all models presented an R^2^ greater than 0.900, which means that the adsorption process is complex, and that more than one mechanism might take place. The parameters *h*, *C*, and *a* are related to the initial adsorption speed and demonstrated that the initial adsorption happens more quickly for walnut and chestnut shells. The parameter *b* was highest for chestnut shell (7.67), followed by walnut (6.84), burnt wood (5.59), and wood (4.44), which means that the available adsorption surface for the sorbates is higher for chestnut and lower for wood.

## 4. Discussion

### 4.1. Characterization of Materials

The specific surface areas obtained by the BET method for the various materials were quite comparable for walnut shell, chestnut shell, and burnt wood, averaging around 4 m^2^ g^−1^. This is notably higher than the 1.74 m^2^ g^−1^ recorded for regular wood.

The partial combustion of wood resulted in an increased specific surface area, a phenomenon frequently observed during biochar production [33]. Indeed, thermal processes are employed to enhance the specific surface area of biomass, thereby improving adsorption capacity. For instance, the BET surface area, pore diameter, and total pore volume of a hydroxyapatite-biochar nanocomposite were found to be 126.41 m^2^ g^−1^, 9.76 nm, and 0.309 cm^3^ g^−1^, respectively, whereas biochar derived from rice straw exhibited values of 7.15 m^2^ g^−1^ for surface area, 11.34 nm for pore diameter, and 0.020 cm^3^ g^−1^ for total pore volume [34]. These values are higher than those obtained for the materials examined in this study; however, at 26.54 nm, wood has a larger pore size. Larger micropores typically facilitate multilayer adsorption mechanisms [35], which were observed during the sorption process on wood, whose best fit to the Freundlich model indicates multilayer adsorption. An activated carbon produced from apricot waste at 400 °C yielded a specific surface area of 3.97 m^2^ g^−1^ [36], which is similar to the specific surface areas found for the materials analyzed in this research. The specific area of burnt wood is similar to that of biochar prepared from rice straw at 400 °C (4.4 m^2^ g^−1^) [37]. However, a higher value of 12.378 m^2^ g^−1^ was obtained for biochar produced with sugarcane bagasse [38].

The diffractograms (from PXRD) revealed differences in the degree of order within the materials. While some polycrystalline domains were evident in the wood-based samples, burnt wood appeared completely amorphous, indicating the substantial degradation of its ordered structure due to thermal modification.

Regarding the SEM analysis, the images obtained did not reveal distinctive morphological features, although all the materials presented irregular surfaces. However, bio-adsorbents produced by *Phytolacca ameritcana* L. biomass exhibited a relatively smooth surface with small pores and an irregular morphological structure [39]. Another study with garlic peel showed a surface that was porous, rough, and irregular [40]. 

These results suggest that walnut and chestnut shells, with their relatively high surface areas and good pore volumes, may be promising candidates for adsorption applications.

### 4.2. Initial Concentration of Pb Ions

The initial ion concentration plays a crucial role as a driving force in overcoming the mass transfer resistance of metal ions with aqueous phase and solid bio-adsorbents [40]. In this study, a decreasing tendency in the removal was observed when increasing the initial concentration of lead. A similar pattern was observed when testing activated carbon and kaolin, in the removal of Pb and Zn. By increasing the initial concentration of both metals, the adsorption capacity decreased [41].

Similarly, when using olive stones to adsorb Pb, the percentage of sorption decreased up to 60% as the initial concentration increased from 2 to 5 mg L^−1^. Also, a higher removal rate of 94.5% for 1 mg L^−1^ was identified, which is lower than the maximum removal rate achieved in the present study (99%) [42]. Similarly, when pine bark was employed in Pb removal with initial concentrations between 50 and 1000 mg L^−1^, the removal decreased when the initial concentration of Pb increased in the solution [43].

Another study used magnetite nanoparticles to remove lead ions. The effect of the initial concentration was studied from 25 to 200 mg L^−1^. The percentage of adsorption decreased when increasing the concentration of Pb, with the highest adsorption around 99%, at 20 mg L^−1^ [44]. Similarly, *Michelia figo* was tested in the removal of Pb, Cu, and Cd, and it was reported that the removal efficiency decreased at higher initial ion concentrations. This was explained as being due to the saturation of functional groups in the biosorbent surface [45].

In contrast, in the use of *Moringa oleifera* seeds and *Musa cavendish* peels in the adsorption of Pb, Ni, and Cd, both materials showed an increase in removal when increasing the concentration of Pb, until 60 μg L^−1^ [46].

### 4.3. Adsorption Isotherms

The adsorption capacity of garlic peel increased when the Pb ion concentration increased from 1 to 50 mg L^−1^. An adsorption capacity of 51 mg g^−1^ was achieved, similar to the maximum adsorption capacity found for walnut and chestnut in this study (around 47 and 44 mg g^−1^, respectively). The authors related this factor to sufficient adsorption sites available in the sorbent surface for the retention of ions [40].

Both the Langmuir and Freundlich models worked well for all adsorbents, with correlation coefficients higher than 0.900. Similarly, both the Langmuir and Freundlich isotherms worked well to describe the adsorption of Cd and Pb onto industrial chili (*Capsicum annuum*) seed waste, although the Langmuir model was chosen to describe the equilibrium data [47]. Likewise, the Langmuir model better described the adsorption of lead onto activated carbon developed from apricot stone (R^2^ = 0.999), but the correlation coefficient for the Freundlich model (R^2^ = 0.984) was also high [48].

The adsorption of Pb by *Moringa oleifera* seeds and *Musa cavendish* best fit the Freundlich model, with R^2^ values between 0.97 to 0.99 [46]. In contrast, when the removal of Pb by activated carbon prepared from coconut shells was tested, the Langmuir model described the isotherm of adsorption best, with R^2^ = 0.9955–0.9996 [49]. In the same way, the adsorption of Pb, Cd, and Mn onto pruning-derived biochar (*Ligustrum vulgare*) was tested. The results showed that the Langmuir model (R^2^ = 0.99) fit the experimental data better than the Freundlich model (0.82 ≤ R^2^ ≤ 0.93) [50].

Relating to *qmax*, the values found in this work for walnut and chestnut shells were greater than the values of 25.91 mg g^−1^, 21.20 mg g^−1^, and 37.88 mg g^−1^ reported for the removal of Pb by banana, orange, and lemon peels, respectively [51]. Furthermore, the maximum adsorption capacity for Pb by activated carbon from coconut shells was 13.855 mg g^−1^ [50], similar to the values found for wood (around 16.6 mg g^−1^). The *n* values obtained in this work ranged between 1.205 and 2.289, showing a good interaction between adsorbent materials and lead ions. Similar results were presented for activated carbon prepared from coconut shell, with *n* = 1.8–3.7 [49].

The Langmuir model is based on monolayer sorption and the homogeneity of adsorption sites, while the Freundlich model is based on multilayer sorption and the heterogeneous distribution of adsorption sites [4,52,53]. Therefore, we can conclude that the adsorption of lead onto walnut, chestnut, and burnt wood is ruled by monolayer sorption, while for wood, multilayer sorption seems to better describe the adsorption process. This can be due to differences in the pore size, as earlier studies have shown that larger-sized micropores generally lead to a multilayer adsorption mechanism [54,55], and wood has the highest pore size.

The Langmuir model describes chemical adsorption, which is more stable than physical adsorption, depending on active sites and functional groups, while physical adsorption directly depends on adsorbent surface and porosity [19]. In this study, the two kinds of adsorption mechanisms may have occurred, as both models worked well to describe the sorption isotherms. Nonetheless, considering the good correlations presented by walnut, chestnut, and burnt wood with the Langmuir model, the chemical mechanism may be the most significant.

### 4.4. Adsorption Kinetics

The steps for determining the adsorption rate include the diffusion process, chemical reactions, and particle diffusion. Intraparticle diffusion describes adsorption that occurs mostly by diffusion mechanisms. In this study, diffusion might play an important role in the adsorption process, because the model presented a good correlation (higher than 0.900), even though the data fit the PSO model better for all the lignocellulosic materials tested, with the correlation coefficient (R^2^) ranging from 0.998 to 1.000.

Similar results were presented using treated green coconut shell, with R^2^ = 1.000 [56], and sugarcane bagasse biochar, with an R^2^ around 0.99 [38]. A best fit for the PSO model was also reported for lead adsorption on carbon-coated monolith, with an R^2^ from 0.990 to 0.998 [57]. Also, the PSO model better described the kinetics of adsorption of Pb, Cd, and Mn by pruning-derived biochar (*Ligustrum vulgare*), with R^2^ = 0.99, higher than those presented for the PFO model (0.73 ≥ R^2^ ≥ 0.98) [50].

The PFO model is characterized by physisorption, and the PSO, by chemisorption mechanisms [4]. Chemical sorption occurs through chemical bonds at specific functional groups that are irreversible. Previous studies attributed Pb removal to ion exchange reactions [14]. Additionally, it was identified that electrostatic interactions are responsible for the adsorption of Pb ions from water using zeolitic imidazolate frameworks. However, other forces, such as ion exchange, coordination, acid–base, and π–π interactions, are also responsible for the adsorption of Pb [19].

According to the Elovich model, which describes heterogeneous chemisorption, the sorption process only occurs on specific sites, and there is interaction between the adsorbed ions and the energy of adsorption [58]. The good correlation demonstrated by the Elovich model, < 0.90, reinforces the predominance of chemical processes in the sorption of Pb ions onto the lignocellulosic materials tested.

Thus, we can conclude that the adsorption of Pb ions onto walnut and chestnut shells, wood, and burnt wood occurs mainly by chemical forces.

## 5. Conclusions

Our results reinforce the potential applicability of lignocellulosic residues as bio-adsorbents for the removal of heavy metals, especially lead. In addition to strengthening the circular economy, the use of cellulosic byproducts in wastewater treatment has the added value of being more economically and environmentally advantageous than traditional methods.

All the byproducts tested showed a high capacity for lead adsorption, with maximum removal rates of 99% for walnut shells, chestnut shells, and pine wood, and 97% for burnt pine wood, and with low concentrations of lead ions that decreased as the concentration increased.

The adsorption process shows to be complex, given that both the Langmuir and Freundlich isotherm models presented good correlations (R^2^ > 0.90), indicating that adsorption can be monolayer or multilayer, depending on the conditions of the environment and the adsorbent material applied.

All the kinetic models showed a good correlation. However, the good fit of the PSO kinetic model, with an R^2^ between 0.998 and 1.000, indicates that the lead adsorption onto the materials is mostly chemisorption.

The results prove that the materials tested can be successfully applied as bio-adsorbents to remove lead ions from water, enabling more sustainable environmental remediation.

## Figures and Tables

**Table 1:** Isotherm parameters of Pb adsorption onto walnut shell, chestnut shell, wood, and burnt wood.

Models	Parameters	Walnut	Chestnut	Wood	Burnt Wood
Langmuir	*qmax* (mg g^−1^)	47.393	44.444	16.639	19.084
	K_L_ (L mg^−1^)	0.070	0.319	1.295	0.345
	R^2^	0.999	0.992	0.970	0.991
Freundlich	K_F_ (mg^1−n^L^n^ g^−1^)	2.990	7.483	8.002	3.621
	*n*	1.205	1.648	1.545	2.289
	R^2^	0.993	0.967	0.991	0.946

**Table 2:** Experimental results found for PFO and PSO kinetic models.

	Pseudo-First-Order				Pseudo-Second-Order				
	*k*_1_ (L min^−1^)	q_e_ calc (mg g^−1^)	q_e_ exp (mg g^−1^)	R^2^	*k*_2_ (g mg^−1^.min)	*h* (mg g^−1^.min)	q_e_ calc (mg g^−1^)	q_e_ exp (mg g^−1^)	R^2^
Walnut	2.50 × 10^−3^	0.546	4.84	0.956	2.46 × 10^−2^	0.575	4.84	4.84	1.000
Chestnut	2.20 × 10^−3^	0.675	5.12	0.966	2.13 × 10^−2^	0.527	4.98	5.12	1.000
Wood	1.90 × 10^−3^	1.156	4.25	0.977	1.07 × 10^−2^	0.169	3.98	4.25	0.998
Burnt wood	1.80 × 10^−3^	0.907	3.85	0.971	1.47 × 10^−2^	0.191	3.61	3.85	0.999

**Table 3:** The parameters for the kinetic sorption data using the Elovich and intraparticle diffusion kinetic models.

Elovich				Intraparticle Diffusion		
	*a*	*b*	R^2^	*C*	*Kdif*	R^2^
Walnut	9.94 × 10^10^	6.84	0.956	4.23	0.019	0.924
Chestnut	1.05 × 10^12^	7.67	0.990	4.37	0.021	0.942
Wood	1.00 × 10^4^	4.44	0.905	2.99	0.035	0.978
Burnt wood	1.09 × 10^5^	5.59	0.908	2.84	0.027	0.970

## Data Availability

The original contributions presented in this study are included in the article. Further inquiries can be directed to the corresponding authors.
